# Supplementary material for: A New Chemotactic Mechanism Governs Long-Range Angiogenesis Induced by Patching an Arterial Graft into a Vein
Source: Int J Mol Sci. 2022 Sep 23;23(19):11208. doi: 10.3390/ijms231911208 (PMC9569559; doi:10.3390/ijms231911208)
Supplement: Supplementary file 1 [file ijms-23-11208-s001.zip › ijms-1896719-supplementary.pdf]

Figure S1

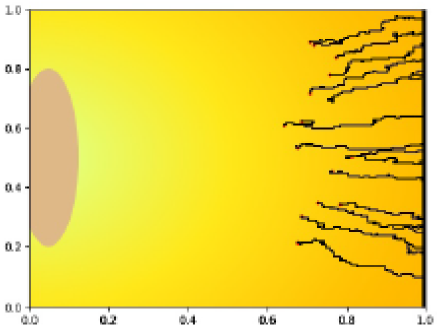

(a) $t = 1$

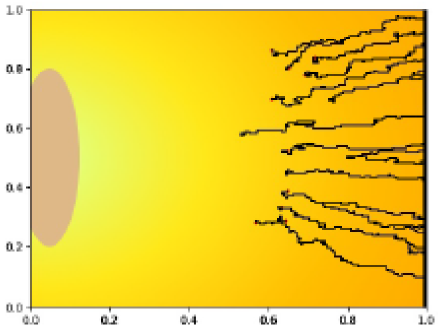

(b) $t = 2$

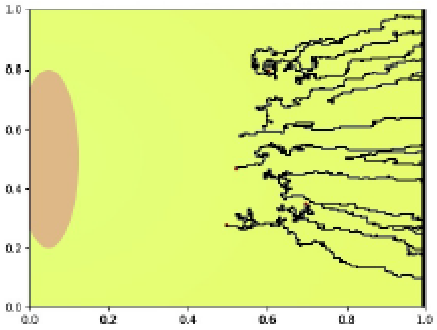

(c) $t = 7$

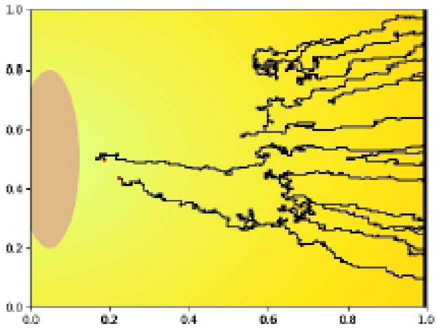

(d) $t = 10$

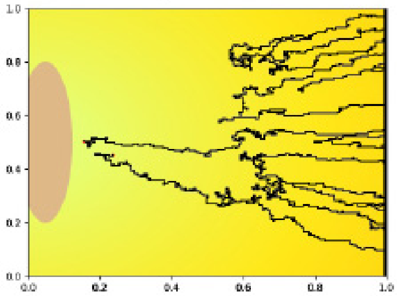

(e) $t = 11$

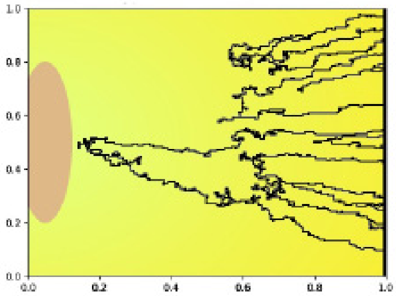

(f) $t = 14$

Figure S2

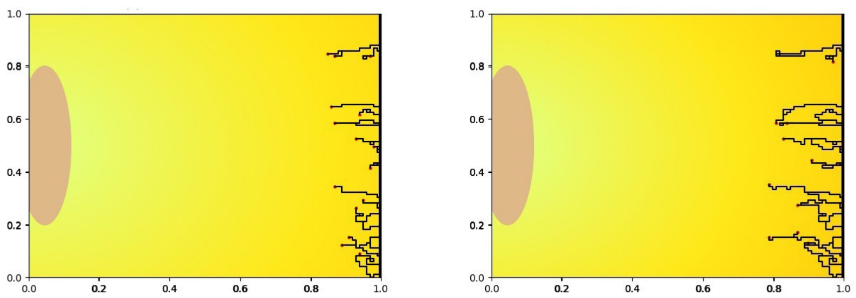

(a) $t = 1$ (b) $t = 2$

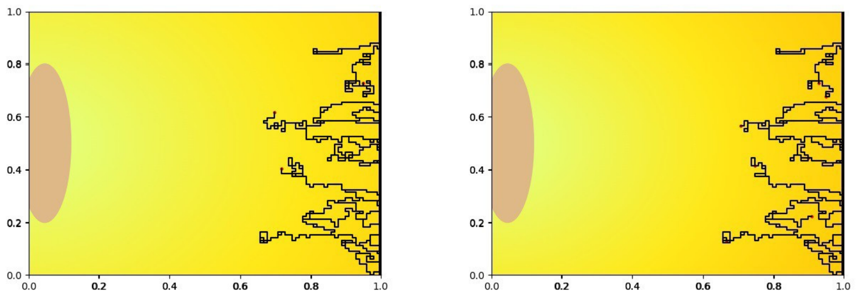

(c) $t = 7$ (d) $t = 10$

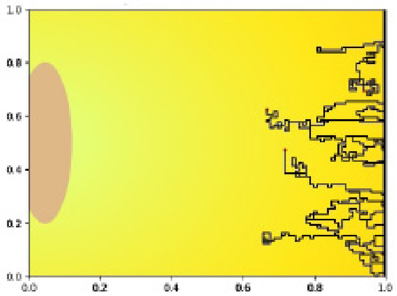

(e) $t = 11$

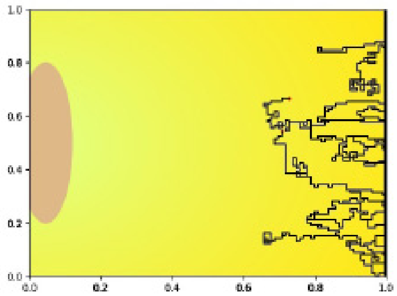

(f) $t = 14$

Figure S3

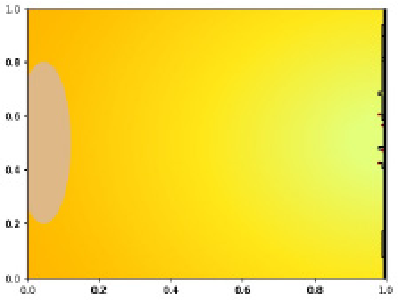

(a) $t = 1$

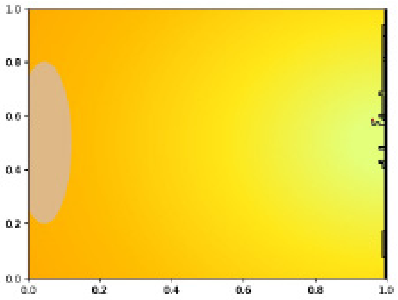

(b) $t = 2$

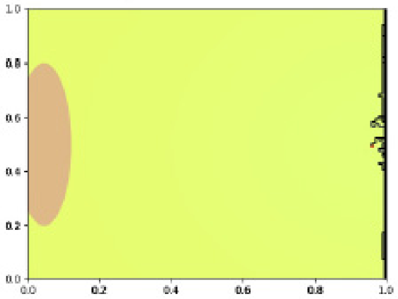

(c) $t = 7$

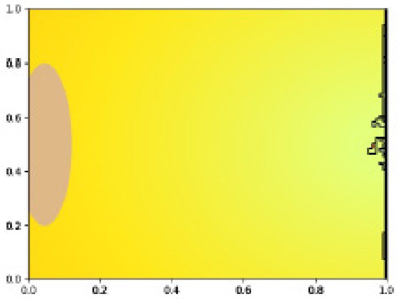

(d) $t = 10$

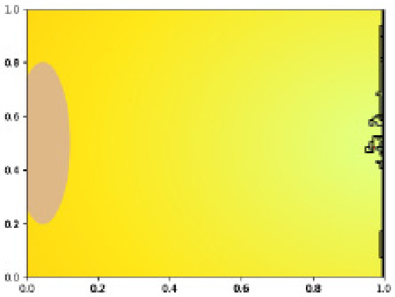

(e) $t = 11$

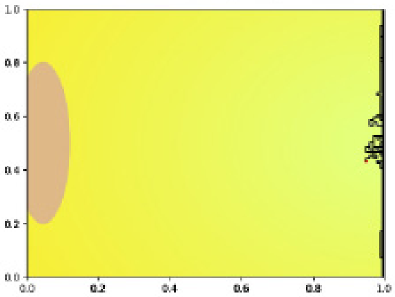

(f) $t = 14$

Figure S4

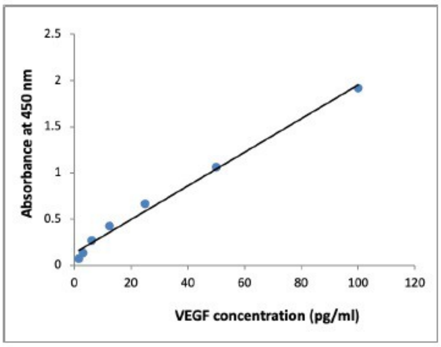

Figure S1. The simulation results of the new blood vessel growth with the background of VEGF concentration level, captured on day $1, 2, 7, 10, 11$, and 14 with the same parameters except $h = 0.005$.

Figure S2. The simulation results of the new blood vessel growth with the background of VEGF concentration level, captured on day $1, 2, 7, 10, 11$, and 14 $x, y \in (0, 1)$, $a = 1.0$, $b = 0.39$, $\sigma = 0.06$, $\gamma = 0.15$, $x_p = 1$, and $h = 0.01$.

Figure S3. The simulation results of the new blood vessel growth with the background of VEGF concentration level, captured on day $1, 2, 7, 10, 11$, and 14 $x, y \in (0, 1)$, $a = 0.6$, $b = 0.39$, $\sigma = 0.23$, $\gamma = 0.15$, $\epsilon = 2.9$, $x_p = 0$, $y_p = 0.5$, $x = 1$, and $y = 0.5$ and $h = 0.01$.

Figure S4. The ELISA signal (absorbance at 450nm) is proportional to the concentration of VEGFA.
